# Drought and Freezing Compromise Woody‐Plant Functioning in High Mountain Ecosystems Under Mediterranean Climate: The Case of *Bencomia exstipulata*

**DOI:** 10.1111/ppl.70965

**Published:** 2026-07-03

**Authors:** Beatriz Fernández‐Marín, Alicia Victoria Perera‐Castro, Pavel Brito‐Gutiérrez, Miren Irati Arzac, Franciso Javier Díaz Peña, Andrea Ganthaler, Celine García‐Rodríguez, José Manuel Laza, Vanesa Cristina Luis, June Hidalgo, Stefan Mayr, Gilbert Neuner, Marcos Adrian Ruiz‐Medina, José Ignacio García‐Plazaola, Jaime Puértolas, Águeda María González‐Rodríguez

**Affiliations:** ^1^ Department of Plant Biology and Ecology University of the Basque Country (UPV/EHU) Leioa Spain; ^2^ Department of Botany, Ecology and Plant Physiology Universidad de La Laguna La Laguna Canary Islands Spain; ^3^ Department of Biology Universitat de les Illes Balears Palma Spain; ^4^ Institute for Plant Molecular and Cell Biology (IBMCP), CSIC‐UPV Valencia Spain; ^5^ Departamento de Biología Animal, Edafología y Geología, Facultad de Ciencias Universidad de La Laguna (ULL) San Cristóbal de La Laguna Spain; ^6^ Department of Botany University of Innsbruck Innsbruck Austria; ^7^ Department of Chemical Physic University of the Basque Country (UPV/EHU) Leioa Spain

## Abstract

*Bencomia exstipulata* is a broad‐leaved evergreen shrub endemic to the high‐altitude harsh landscapes of the Canary Islands. Whether the extremely reduced distribution of its wild specimens (at around 2000 m a.s.l. in the National Parks of ‘El Teide’ and ‘Caldera de Taburiente’) responds to specific microclimatic needs remains undetermined. None of its ecophysiological aspects has been evaluated to date. To fill this gap of knowledge, we have (1) characterised its leaf phenology and physiology, (2) evaluated its tolerance to drought at leaf and xylem levels, and (3) investigated its response to freezing stress at biophysical and photochemical levels. Our results revealed that *B. exstipulata* has a fast leaf turnover with high rates of photosynthesis and stomatal conductance and high xanthophylls per chlorophyll ratios. Leaves had thin cuticle, high minimum leaf conductance, and encrypted and abaxial stomata. Adult leaves lost 50% of their rehydration capacity at around 45% relative water content, and stem xylem was relatively vulnerable to embolism (with −3 MPa of water potential at 50% loss of conductivity), but no native embolism was found. Under freezing temperatures, leaves showed a supercooling strategy (ice nucleation at −14°C) and low photoprotective responsiveness. We conclude that *B. exstipulata* lacks clear adaptations to drought, has high constitutive photoprotection, low photoprotective responsivity, and a supercooling strategy to face freezing. In its native montane ecosystem, with a Mediterranean‐type climate, severe drought could induce significant xylem‐embolism, while severe freezing could lead to irreparable leaf damage, being both risks potentially lethal in the mid‐ to long term.

## 1 Introduction

*Bencomia exstipulata* Svent., also known as ‘Rosal del Guanche’, is a remarkable shrub (to small tree) endemic to the high‐altitude rocky landscapes in the Canary Islands. It was formally described in 1960 by the Spanish‐Swedish botanist Eric Ragnor Sventenius, based on specimens collected in the high‐mountain areas of Tenerife and La Palma islands (Sventenius 1960). Despite its conspicuousness, the species had not been previously mentioned in relevant botanical expeditions, that is, by von Humboldt (1799) nor by von Buch and Smith (1819, 1825), likely due to the restricted distribution of the species, which grows on cliffs and steep slopes (Renner et al. 2023). Four species represent the genus in Macaronesia, with distinct ecological zones: *B. caudata* Webb & Berth. (Madeira, Tenerife, La Palma and Gran Canaria, 500–1200 m a.s.l.), *B. brachystachia* Svent. ex Nordborg (endemic to Gran Canaria, 1100–1700 m a.s.l.), *B. sphaerocarpa* Svent. (endemic to El Hierro, 400–1200 m a.sl.) and *B. exstipulata* (endemic to Tenerife and La Palma, 2000–2500 m a.s.l.), which represents the tallest woody angiosperm inhabiting the shrub habitat over the timberline at the slopes of Mt. Teide National Park (3715 m a.s.l.) (Marrero et al. 2019). Interestingly, *Bencomia* is taxonomically included in the Agrimonieae tribe (Rosaceae) (Zhang et al. 2017), the corresponding tribe of the tree species growing at the highest elevation on earth: *Polylepis tarapacana* (Braun 1997; Liberman‐Cruz et al. 1997). Over the past 20 years, *B. exstipulata* has been the subject of conservation actions aimed at lowering its extinction risk (Marrero et al. 2019). In 2019, only 57 wild individuals remained in Teide National Park and 117 in Taburiente National Parks (González‐Pérez et al. 2009; Marrero et al. 2019). Demographic, reproductive and genetic aspects of the species have been extensively studied (Helfgott et al. 2000; González‐Pérez et al. 2004, 2009; Marrero et al. 2019) and efforts have focused on seed banking and the reinforcement of natural populations using nursery‐grown individuals (Marrero et al. 2015, 2019). These measures have led to a notable increase in both population size and area of occupancy (Marrero et al. 2015, 2019). Consequently, its IUCN status was upgraded from Critically Endangered to Vulnerable (Bañares Baudet et al. 2011) corroborated in 2015 (Marrero et al. 2015). However, the species remains listed as Endangered in both the Canary Islands and the Spanish national catalogues of threatened species (BOIC. 2010; BOE 2011) and, notably, none of its ecophysiological features have been evaluated to date, even when a profound ecophysiological characterisation can improve our understanding of the species' ecological amplitude and its response to the current and future conditions in the particular environment where it grows. Together with herbivory pressure, the physical limitations imposed by the harsh conditions of the high elevations of the Canary Islands, exacerbated by global change, are likely the main limitations for successful conservation of this species.

Teide National Park, centred on the 3715 m Teide volcano of Tenerife Island, hosts a high‐elevation ecosystem with a distinctly Mediterranean‐type climate. This includes a strong summer drought and frequent sub‐zero temperatures throughout the year, conditions that are uncommon at similar latitudes (Alday Echechipia et al. 2025; MiTECO 2025). Above 2000 m, cloudiness sharply disappears and rainfall decreases dramatically due to a temperature and humidity inversion layer that forms on the windward slopes (~1200 m a.s.l.), resulting in mean annual precipitation of < 350 mm (Fernández‐Palacios and Nicolás 1995; de Canarias and de Tenerife 2019, 2020, 2021). This annual precipitation value is highly variable: for example, the last available annual report showed 239 mm (PN del Teide 2021) and was as low as 50 mm in 2012 (Martin‐Esquivel and Perez‐Gonzalez 2019). The soils, developed over highly permeable volcanic parent materials (e.g., fragmented lavas, tephra, and scoriaceous deposits), are usually coarse‐textured and skeletal, resulting in high porosity and rapid drainage, which promote prolonged soil dry periods during the warm season (Brito et al. 2013). Air temperatures can drop below 0°C during most of the year and rise above +25°C in summer, with surface‐level extremes reaching −10°C to +50°C, and are strongly influenced by microtopography and microclimate (López Díez et al. 2021; Alday Echechipia et al. 2025). Above the cloud layer, the pine forest is replaced by open shrub communities with few dominant taxa adapted to this semi‐arid ecotone and a total of 168 alpine species (del Arco and Rodríguez 2018; González‐Rodríguez et al. 2020) that must be at least to some extent adapted to summer drought and cold. Thus, although it could be expected that *B. exstipulata* is also adapted to this highly demanding environment, whether the extremely reduced distribution of its wild specimens is due to specific microclimatic needs, remains unknown. In this sense, drought and freezing stresses are expected to be two of the most determining factors.

Water scarcity is a major constraint for plants, impairing photosynthetic function and affecting water relations at both leaf and xylem levels (Tyree and Zimmermann 2002, Boisvenue and Running 2006; Fernández‐Marín et al. 2016, 2024). Stomatal conductance (g_s_) plays a central role in drought response by balancing carbon uptake and water loss. While some species maintain gas exchange until late dehydration stages (anisohydric behavior), others restrict g_s_ early to preserve xylem function and maintain water potential (isohydric strategy) (Tardieu and Simonneau 1998). With increasing dehydration, cuticular water loss becomes increasingly relevant, and minimum leaf conductance (g_min_) provides an estimate of water leakage once stomata are fully closed (Duursma et al. 2019). Although g_min_ values are often low, small differences can substantially influence leaf desiccation rate. Under severe dehydration, leaves may reach a threshold beyond which they lose the capacity to recover, known as loss of rehydration capacity (LRC) (Trueba et al. 2019), a useful marker of tolerance to water deficit across species (John et al. 2018). At the whole‐plant level, resistance to xylem embolism determines the hydraulic safety and the risk of losing conductive capacity under drought (Choat et al. 2010). Species vary widely in their xylem resistance, typically expressed as the water potential causing 50% loss of hydraulic conductivity (Ψ_50_), with consequences for their survival of drought events (Delzon and Cochard 2014). The specific hydraulic conductivity (k_s_) additionally determines the hydraulic efficiency of water transport in the xylem (Tyree and Ewers 1991). Both hydraulic safety and efficiency are strongly related to xylem anatomical characteristics. Together, these traits are generally related to a plant's position along the safety versus efficiency correlation, with some species falling at the low efficiency and low safety corner of the relationship (Gleason et al. 2016). Thus, generally although with exceptions, these traits shape species' resilience to drought, and their phenotypic and genotypic plasticity is important with respect to climate change. All these aspects are completely unknown for *B. exstipulata* .

Freezing stress poses a dual threat to plant tissues through both physical and physiological processes. Ice formation within cells is typically lethal, prompting species to either avoid freezing via supercooling or tolerate extracellular ice while avoiding intracellular ice formation (Sutinen et al. 2001; Arora 2018). The efficiency of these strategies depends on temperature thresholds and tissue properties. Anatomical traits, such as ice barriers in leaf or bud tissues, can delay or restrict ice propagation, protecting sensitive structures (Bertel et al. 2021) and can be studied through Infrared Differential Thermal Analysis (Hacker and Neuner 2007). At the physiological level, freezing can impair photosynthetic performance, especially under high light (Verhoeven et al. 2018). Plants counter this with enhanced photoprotective responses, including the activation of the xanthophyll cycle and thermal energy dissipation (Fernández‐Marín, Neuner, et al. 2018; Fernández‐Marín et al. 2021). Changes in chlorophyll fluorescence parameters, such as the photochemical efficiency of PSII (Fv/Fm), often reflect these protective adjustments. Thus, some species down‐regulate the photochemical efficiency throughout winter (chronic winter photoinhibition), while others do it in a short (< 12 h), reversible way (dynamic photoinhibition) (Míguez et al. 2015). In some cases, tissues enter a vitrified or ‘glassy’ state, where high viscosity and reduced molecular mobility limit enzymatic activity and structural damage during freeze–thaw cycles. Glass transition temperature (T_g_) is a very informative parameter in that sense that can be estimated through dynamic mechanical thermal analyses (DMTA) (Fernández‐Marín, Neuner, et al. 2018; Georgieva et al. 2022). Additionally, most plants' tolerance to freezing varies along seasons due to acclimation and de‐acclimation processes (Sklenar 2017, González‐Rodríguez et al. 2021). This suite of structural, biochemical and biophysical adaptations ultimately determines a species' cold resilience and survival in alpine or subalpine environments. Their success is particularly critical during early plant and leaf developmental stages, with usually higher vulnerability to frost events, limiting the seedling recruitment rates (Marcante et al. 2012). All these physiological and biophysical features remain entirely unexplored in *B. exstipulata*.

In the current context of climate change, mountain environments, and very importantly, those in oceanic islands characterised by their high endemicity index, are subjected to drastic and quick changes. In Teide Mountain in particular, increasing evidence points to climate warming and introduced herbivores as relevant disruptors of the alpine plant community (Cubas et al. 2018; Martín‐Esquivel et al. 2020). Among abiotic factors, increasing temperature is currently considered the main driver of shifts in vegetation with a remarkable measured warming of 1.7°C ± 0.6°C per decade in the period 1975–2015 (Martin‐Esquivel and Perez‐Gonzalez 2019; Martín‐Esquivel et al. 2021). Additionally, in terms of precipitation, a drier trend has been determined in the last decades. Thus, not only is the yearly cumulative precipitation, on average, lower than in the previous century, but also the length of the humid period is shorter (Martin‐Esquivel and Perez‐Gonzalez 2019). Last, but not least, being a high mountain ecosystem with very particular climatic conditions above the temperature and humidity inversion layer (beyond the clouds) is a very dry environment with high probability of freezing events all along the year, the extent and frequency of which could also be altered in the context of climate change (Alday Echechipia et al. 2025). To what extent *B. exstipulata* already grows close to its physiological limit for drought and subzero temperatures in this perspective of climate change remains undetermined.

Considering all these aspects, the aim of this study was to identify the key ecophysiological traits that may induce a failure in the strategy of *B. exstipulata* to persist in high‐elevation environments facing drought and subzero temperatures under a Mediterranean climate and on a climate change scenario. Specifically, we (1) characterised its leaf phenology and physiology, (2) evaluated its resistance to drought at both the leaf and xylem levels, and (3) investigated its response to freezing stress, including biophysical and photochemical processes. We hypothesise that *B. exstipulata* may have strong resistance to hydraulic failure and efficient mechanisms protecting cellular and photochemical integrity under freezing conditions.

## 2 Materials and Methods

### 2.1 Study Site and Experimental Design

The study was conducted in El Portillo (El Teide National Park, 28.30° N, 16.57° W, 2060 m a.s.l.) under field conditions in 21 healthy and adult individuals of approximately 2 m height and 15–20 cm stem diameter. Leaf phenology was evaluated in all of them from February to December 2021. From these 21 plants, five individuals were selected, and xylem hydraulic properties were analysed. Physical aspects of adult leaves at subzero temperatures were also analysed in 5 individuals. Thereafter, three leaf‐stages were differentiated: young (developing and folded), adult (fully expanded and fully green), and senescent (fully expanded but yellowish). Leaf anatomy, photosynthetic pigments, gas exchange and soluble carbohydrate were measured separately in the three leaf age‐types of five to six individuals, depending on the parameter. Loss of rehydration capacity was evaluated in the three leaf age‐types of 20 individuals. Additionally, 10 one‐year‐old potted seedlings were used for freezing experiments under controlled and semi‐controlled (outdoor) conditions. Seedlings were grown outdoors at El Portillo Botanical Garden nursery, and then transferred to the University of Innsbruck (UIBK) (47.27° N, 11.38° E, 600 m a.s.l.) in December 2019 for the freezing experiments. From these 10 seedlings, four to eight were used, depending on the parameter analysed. Under controlled freezing conditions, ice propagation pattern, changes in photochemical efficiency (Fv/Fm) and photosynthetic pigments were evaluated. Under outdoor seminatural conditions, the variation in predawn Fv/Fm was monitored. All physiological measurements of this study were conducted in three seasons: physiology and biophysics of leaves against freezing stress were evaluated in winter (December 2019–March 2020), gas exchange, photosynthetic pigments, leaf anatomy and loss of rehydration capacity were evaluated in spring (June 2021 and 2025), drought resistance of stem xylem was analysed at the end of summer (September 2021).

### 2.2 Leaf Phenology

*B. exstipulata* has odd‐pinnate leaves, with 5–7 leaflets. Leaf number per branch and leaf area were monthly monitored during 1 year in 21 adult plants. One south‐oriented branch and one sprouting leaf were labelled and monitored in each plant. Leaves were considered for the phenological study until they were naturally shed from the branches. This species lacks leaf buds. Thus, the leaf phenophases studied were essentially: unfolded young leaves, fully expanded leaves and green‐yellow transitioning leaves (the transitions among them were also monitored). Non‐functional (fully dried and dead leaves) were also monitored until naturally detached. The number of leaves was counted in the field, while photographs of the labelled leaves were analysed with ImageJ v1.5 software (Wayne Rasband, National Institutes of Health) in the lab. Blade area was estimated as the sum of the areas of the leaflets, excluding the rachis.

### 2.3 Leaf Anatomy

One leaf per type and per plant was collected from 5 different individuals in June 2021 and processed as follows. A small piece of the apical leaflet was cut and immediately fixed in FAA (100 mL FAA = 90 mL 70% ethanol +5 mL acetic acid 96% + 5 mL formaldehyde solution 37%) for 48 h. Samples were then rinsed with distilled water and dehydrated by sequentially immersing the sample in increasing concentrations of ethanol. Leaf pieces were then embedded in paraffin and sectioned at 6 μm thickness with a rotary microtome MT.5505 (PCE Iberica S.L.). All sections were de‐waxed with a xylene series, stained with safranin and counterstained with fast green (Gerlach 1984). Finally, samples were preserved using the EUKITT mounting medium (O. Kindler GmbH). Leaf anatomy was examined with a light microscope (AmScope), coupled with a Samsung SM‐G955 camera. Image analysis was conducted in ImageJ v1.5 software (Wayne Rasband, National Institutes of Health, Bethesda, MD, USA) to quantify the cuticle thickness, the % of leaf transversal section occupied by palisade + spongy parenchymae, and the diameter of the bundle sheet.

Five additional leaves were used for measuring leaf mass per area (LMA) and saturated water content (SWC). Individual leaves were photographed with an iPhone 8 and their projected area was estimated with Image J. Saturated weight (SW) was estimated as the weight after overnight incubation in a sealed container with petioles in contact with distilled water. After that, leaves were dried in an oven for at least 48 h, when the desiccated weight (DW) was obtained. LMA was calculated as the ratio between DW and projected area. SWC was calculated as:

$$\mathrm{SWC}=\frac{\mathrm{SW}-\mathrm{DW}}{\mathrm{DW}}$$

### 2.4 Photosynthetic Pigment, Tocopherol and Soluble Carbohydrate Analyses

Six leaves (two per type) were used from five different plants in June 2021: one for photosynthetic pigment and tocopherol analyses and the other for soluble carbohydrate quantification. Samples (4 discs of 4 mm ø, per replicate) were collected at midday in the field, immediately immersed in liquid nitrogen, transported to the lab and then stored at −80°C until analysis of pigments and tocopherols. Leaf discs of one of the two replicates per leaf type and plant were then extracted in cold acetone buffered with CaCO_3_ and finally analysed by HPLC following the method described in (Fernández‐Marín, García‐Plazaola, et al. 2018). The most common photosynthetic pigments (carotenoids: neoxanthin, antheraxanthin, violaxanthin, zeaxanthin, lutein epoxide, lutein, beta‐carotene; and chlorophylls a and b) plus two different tocochromanols (alpha, and delta‐tocopherol) were analysed (see Esteban et al. 2015 and Fernández‐Marín et al. 2017; Fernández‐Marín, García‐Plazaola, et al. 2018 for further information about their typical concentrations and functions). The other sample was immersed in liquid nitrogen in the field, and freeze‐dried in the lab and powdered for carbohydrate quantification. Approximately 0.2 g (exact dry weight recorded) were extracted in 80% ethanol to quantify soluble carbohydrates. Starch residue was hydrolysed with anthrone reagent (0.2% m/v anthrone diluted in 95% sulphuric acid). Both soluble and hydrolysed sugars were determined by spectrophotometry (Shimadzu, UV‐160 A) according to Irigoyen et al. (1992).

### 2.5 Gas Exchange Measurements

Simultaneous gas exchange and chlorophyll fluorescence measurements were performed in the field in late spring (June 2021) with the open system Li‐6400XT (Li‐Cor Inc., Lincoln, NE, USA), coupled to a fluorescence chamber of 2 cm^2^. Light saturated net CO_2_ assimilation (A_N_), stomatal conductance to CO_2_ (g_s_) and yield of photosystem II (фPSII) were recorded at 400 μmol CO_2_ mol^−1^ air, 1500 μmol photons m^−2^ s^−1^ (previously determined to be saturated by semi‐light curve), 25°C of temperature and 20%–50% of ambient humidity in the chamber. Randomised measurements of the three leaf types were performed on five individuals during 9:00–15:00 h of 2 consecutive days of similar environmental conditions [completely sunny, air T ≈ 20°C, relative humidity (RH) 30%]. Photoinhibition at midday was not observed and there was no significant effect of diurnal time in A_N_, фPSII or g_s_. The values of each individual and leaf type resulted from averaging three recordings of different leaves of the same age performed at different diurnal times (*n* = 5 individuals).

Minimum diffusion conductance of water vapour across the epidermis (g_min_) once stomata are closed was measured following Sack and Scoffoni (2011). Five leaves of each type from different plants were collected in the field. Under laboratory conditions, leaf cut ends were sealed with parafilm and leaves were hung from a rack in front of a fan that gently swayed them in order to minimise their boundary layer resistance. Meanwhile, lost leaf mass was monitored for 1 h, until the rate of weight loss was nearly constant. Temperature and humidity were recorded using an open gas exchange system (Li‐6400XT; Li‐Cor Inc.) to calculate the vapour pressure deficit (VPD), which remained constant during dehydration measurement. The transpiration flux of water (E) of each leaf was obtained from the mass‐time relationship and g_min_ was then calculated as g_min_ = E/VPD.

### 2.6 Estimation of Loss of Rehydration Capacity

The apical leaflet of each leaf was used for the analysis of loss of rehydration capacity (LRC) by using *n* = 20 biological replicates per leaf type (i.e., juvenile, adult, senescent) following the procedure of Trueba et al. (2019). Leaves were bench‐dried at 20°C and 65% RH in darkness. Leaf weight and the maximal photochemical efficiency of PSII (Fv/Fm) were measured at turgor 12 h after incubation with the petiole immersed in distilled water at 100% air RH (t_0_), after the desired dehydration (t_Dh_) and after 24 h of rehydration (t_Rh_). At the end of the experiment, the dry weight (DW) of every sample was obtained by oven‐drying for 72 h at 70°C to calculate the leaf relative (to t_0_ water content) water content at t_Dh_ (RWC_Dh_). Chlorophyll fluorescence was measured with a MINI‐PAM (Heinz Walz). The percentage loss of rehydration capacity (PLRC) was calculated as 1 − (RWC_Rh_/RWC_0_) × 100 and the percentage loss of Fv/Fm at dehydration (PLCF_Dh_) as (1 − (Fv/Fm)_Dh_/(Fv/Fm)_0_) × 100. Additionally, we also calculated the percentage loss of Fv/Fm after rehydration (PLCF_Rh_) as (1 − (Fv/Fm)_Rh_/(Fv/Fm)_0_) × 100. These three parameters were plotted against RWC_Dh_ and the best model for each leaf age was fitted according to the methodology described by Trueba et al. (2019). Best model was used to calculate the RWC_Dh_ inducing 50% of the PLRC, PLCF_Dh_, and PLCF_Rh_ (PLRC_50_, PLCF_Dh50_, and PLCF_Rh50_, respectively) and inducing the 10% (PLRC_50_, PLCF_Dh50_, and PLCF_Rh50_, respectively).

### 2.7 Xylem Hydraulics

Embolism resistance was analysed on five branches from five adult individuals at the end of summer (September 2021). Analyses were done with the Cavitron method (Cochard 2002; Cochard et al. 2005) using a 28 cm custom‐built rotor. Samples were cut from branches underwater by re‐cutting branches repeatedly from both sides with a pruning knife. About 5 cm of both sample ends were debarked and sample ends finally trimmed with a sharp wood carving knife (Beikircher and Mayr 2016) before fixing them in the cuvettes of the rotor. Cuvettes were then filled with distilled and filtered (0.22 μm) water containing 0.005% (v/v) (Micropur Forte MF 100F) and rotational speed set to a target xylem pressure (P) of −0.25 MPa. After an equilibration time of about 10 min, the water flow from the upstream to the downstream reservoir was measured based on the observation of the moving meniscus in the reservoir with a high‐resolution camera (Motic 1SP, Motic Deutschland GmbH) and hydraulic conductance (k_h_; m^3^ s^−1^ MPa^−1^) was calculated. Hydraulic conductance was then repeatedly determined at gradually increased rotational speed (and thus decreased P) until −9 MPa at the utmost. Percentage loss of hydraulic conductivity (PLC) was calculated as the ratio of the actual and the first (i.e., maximum) measured k_h_ value and plotted versus the respective P. Curves were fitted for each branch using a Weibull regression and the water potential (Ψ) at 12%, 50%, and 88% PLC (Ψ_12_, Ψ_50_, Ψ_88_) was calculated with the R package fitPLC, R i386 3.2.5 (Duursma and Choat 2017). Values were averaged and the pooled dataset was plotted.

Length and xylem cross‐sectional area of each branch were determined to calculate the specific hydraulic conductivity (k_s_) based on the maximum k_h_ obtained at −0.25 MPa. Five branch samples were used to determine the maximum vessel length to exclude potential artefacts related to the Cavitron technique (Choat et al. 2010; Cochard et al. 2010; Torres‐Ruiz et al. 2014). Branches were connected to an air pump and a pressure (40 kPa) was applied at the basal end, while the distal end was repeatedly shortened by ∼0.5 cm while submerged under water. Branches were re‐cut until a stream of air bubbles indicated that a conduit had been cut open on both sides (Ewers and Fisher 1989; Beikircher and Mayr 2008; Nolf et al. 2014). The remaining length was assumed to be the maximum conduit length. It was 7.05 ± 0.93 cm in *B. exstipulata*, and thus sufficiently below 28 cm (rotor diameter). We additionally stained the conductive xylem area with safranin before and after removal of native embolism by vacuum infiltration (details in Supporting Information: Methods 1) and made a cross‐section (about 15 μm thick) with a sliding microtome (Sledge Microtome G.S.L. 1).

### 2.8 Physical Aspects of *B. exstipulata* Leaves Under Sub‐Zero Temperature

Ice nucleation temperature (T_ice_) and glass transition temperature (T_g_) were evaluated in adult and turgid leaves collected in March 2020 from 5 different plants, following the methods described in Fernández‐Marín, Neuner, et al. (2018) and Georgieva et al. (2022). The T_ice_ was estimated through Differential Scanning Calorimetry using a DSC 822e equipment (Mettler Toledo). This technique measures the amount of energy (heat) absorbed or released by a sample under certain conditions, and that relates to physical/chemical transitions (i.e., exothermic processes such as ice formation). In this study, we subjected leaf tissue to a controlled cooling rate of 0.05°C min^−1^ and determined the temperature at which the exotherm of the ice nucleation event began (T_ice_). The T_g_ was evaluated through Dynamic Mechanical Thermal Analyses using a DMA/SDTA861e equipment (Mettler Toledo). This technique measures the viscoelastic properties of the sample (storage modulus, loss modulus), by applying a controlled, oscillating force while scanning across a range of temperatures. In our DMTA analyses, the shear storage modulus (*G*′) (which estimates a material's ability to store elastic energy) and the shear loss modulus (*G*″) (which estimates its ability to dissipate energy as heat) were measured during the heating run from −60°C to +150°C as is typically used for plant samples (Fernández‐Marín, Neuner, et al. 2018). The delta tangent (Tan δ) was then obtained as *G*″/*G*′. Glass transition temperature (T_g_) was identified as a peak in the Tan δ coincident with a loss in the *G*′ and represents the temperature below which viscosity increases greatly and the probability of enzymatic reactions decreases dramatically (i.e., enters into the glassy state). In this glassy state, plant samples are typically stable and long‐standing.

### 2.9 Physiological Effects of Sub‐Zero Temperatures on *B. exstipulata* Leaves

The physiological effects of sub‐zero temperatures were evaluated on adult leaves of 1‐year‐old seedlings either under controlled or under natural (outdoor) sub‐zero temperatures in December 2019. To evaluate the winter photoinhibitory strategy of the species, eight intact plants were subjected to natural outdoor conditions at the UIBK‐Garden. Their maximal photochemical efficiency of PSII (Fv/Fm) was monitored with a Plant Stress Kit fluorometer (PSK, Opti‐Sciences Inc.) at natural predawn (before 7.30 am) during six subsequent days (8th–13th December 2019). The air temperature surrounding the plants was recorded with an EL USB‐2‐LCD sensor and data logger (Lascar Electronics).

In a second experiment at the UIBK‐lab, detached adult leaves were subjected to controlled freezing to target temperatures of −15°C or −7°C (*n* = 6 biological replicates per treatment and timepoint). In this second experiment, ice propagation through the leaves was assessed through Infrared differential thermal analysis (IDTA) using a digital infrared camera (T650SC, FLIR Systems) and following the protocol described in (Fernández‐Marín, Neuner, et al. 2018; Fernández‐Marín et al. 2021). Briefly, detached leaves were subjected to controlled freezing treatments by cooling rate of 3 K h^−1^, inside a custom‐adapted freezer, and with a wet solution of Ice Nucleation Active (INA) bacteria in touch with the petiole to prevent artificial supercooling (Fernández‐Marín, Neuner, et al. 2018; Fernández‐Marín et al. 2021). In a parallel set of samples, changes in Fv/Fm and photosynthetic pigments were monitored before (t_0_), 16 h after freezing and after 24 h of rewarming (recovery). Chlorophyll fluorescence was measured with a MINI‐PAM (Heinz Walz). Samples for photosynthetic pigment analyses were immediately immersed in liquid nitrogen, and stored at −80°C until HPLC analyses (as described in previous section). All these experiments were conducted in darkness.

### 2.10 Statistical Analyses

Statistical differences among leaf types or treatments for Fv/Fm and metabolite concentrations were assessed by one‐way ANOVA, with Duncan's post hoc test applied after verifying homoscedasticity. Significant differences were considered at *p* < 0.05. These statistical analyses were conducted with IBM SPSS Statistics v.29.0.2.0(20). Gas exchange and xylem vulnerability analyses were conducted in R (the specific packages are already mentioned in the corresponding subsections above).

## 3 Results

### 3.1 Leaf Characterisation: Phenology and Anatomy

**FIGURE 1:**
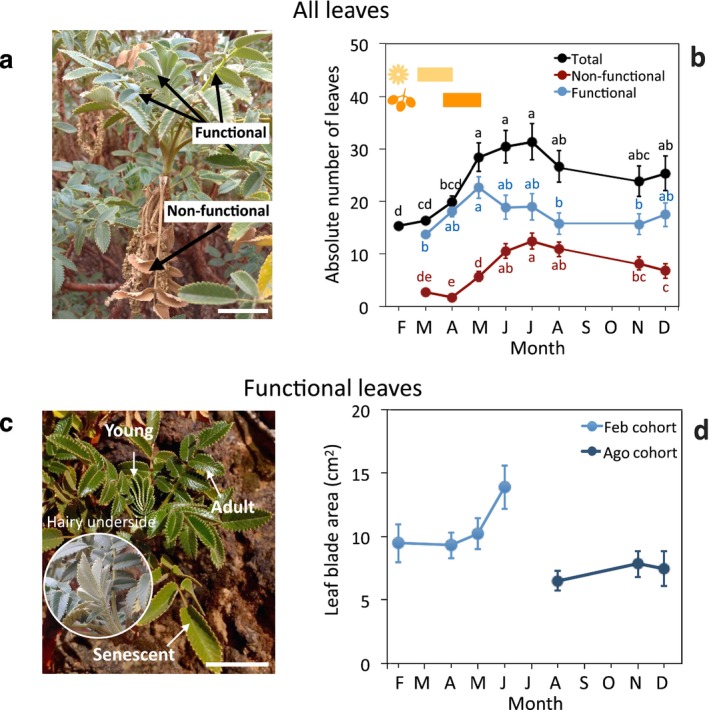
Phenology of *B. exstipulata* leaves from February to December 2021. (a) Representative branch showing the apical arrangement of the functional leaves, and the pendant arrangement of non‐functional (dead) leaves. Scale bar: 2 cm. (b) Change in the number of leaves per branch throughout the year. Total number of leaves, functional leaves and non‐functional leaves are depicted. A total of 21 individuals were assessed. Each data point shows the mean ± SE (*n* = 10–21). (c) The three leaf types of functional leaves studied in this work: Young (still folded leaves), adult (fully expanded and green) and senescent (fully expanded but yellowish). The circular inset shows the densely hairy adaxial side of the leaves. Scale bar: 2 cm. (d) Change in area of individual leaves over the year. Two different cohorts were evaluated, one starting in February (all leaves were dead in July) and the second starting in August 2021. A total of 21 individuals were assessed. Each data point shows the mean ± SE (*n* = 3–21). Photographs (a) and (c) were taken from adult individuals in June 2021.

Phenological monitoring of adult trees enabled us to identify important characteristics of *B. exstipulata*. Leaf arrangement within each branch was apical, with erect functional (green) and pendant non‐functional (brown and dry) leaves (Figure 1a). The number of leaves per branch changed during the year (Figure 1b). Importantly, the maximum number of functional leaves occurred at the end of spring, coinciding with the fruiting period (field observation), and decreased during autumn (from 22.7 ± 2.1 leaves per branch in May to 15.7 ± 2.0 in November, Figure 1b), what may indicate spring as the most demanding season in terms of carbon assimilation. We distinguished three leaf types for the functional (e.g., chlorophyll‐containing) leaves: young, adult and senescent leaves (Figure 1c). All of them were densely covered with hairs, particularly on the abaxial side (Figure 1c inset). Leaf sprouting was continuous throughout the year even in winter (e.g., February, Figure 1d). We monitored two different selected cohorts and determined the average leaf‐blade area during expansion (7.5 ± 1.4 to 13.9 ± 1.7 cm^2^) and the approximate leaf lifespan (5 months, Figure 1d). This leaf lifespan was independent of the time point of leaf emergence (e.g., it was comparable between leaves sprouted in February and those emerged in August).

**FIGURE 2:**
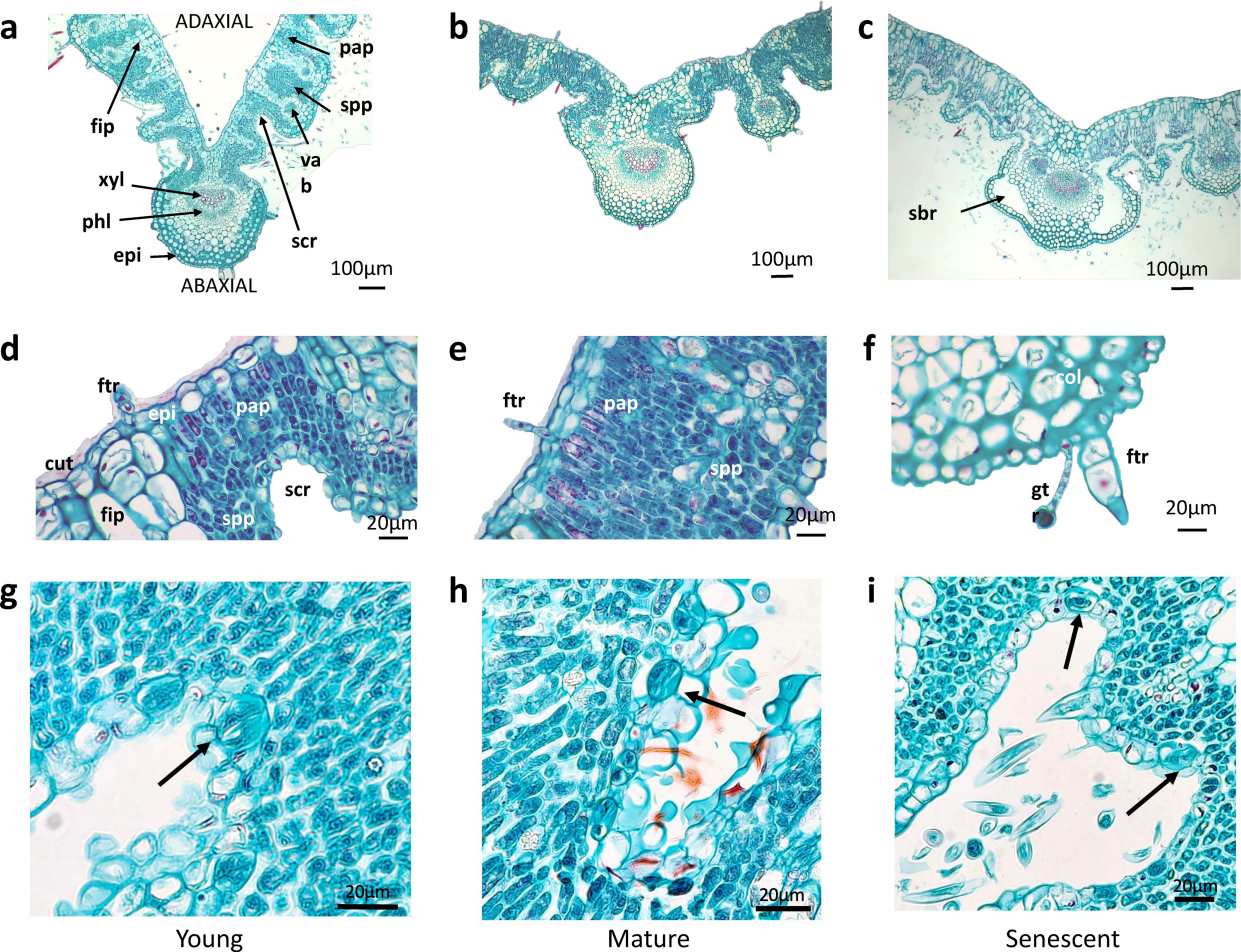
Cross‐section of three leaf types under the light microscope, stained with safranin–fast green staining. (a–c) Whole view of the cross‐sections of young, adult and senescent leaves, respectively. (d) Detail of a cross‐section of a young leaf showing the adaxial cuticle and a stomatal crypt. (e) Detail of a cross‐ section of an adult leaf where the palisade and spongy parenchyma can be seen, with a high number of chloroplasts. (f) Detail of abaxial leaf surface without cuticle and with two different types of hairs. (g–i) Details of stomata (indicated by thick arrows) located in the stomatal crypt. Abbreviations: col., collenchyma; cut, cuticle; epi, epidermis; fip, filling parenchyma; ftr, filiform trichome; gtr, glandular trichome; pap, palisade parenchyma; phl, phloem; sbr, senescent broken tissue; scr, stomatal crypt; spp., spongy parenchyma; tri, trichome; vab, vascular bundle; xyl, xylem.

Anatomy of young, adult and senescent leaves was evaluated under the light microscope. The most remarkable property of *B. exstipulata* leaf anatomy was the presence of abaxial stomatal crypts (Figure 2). On a transversal view, the leaf blade was greatly occupied by two types of photosynthetic parenchyma: the palisade and spongy parenchyma. Surprisingly, an additional tissue type that can be called ‘filler parenchyma’ (without chloroplasts) covered a relevant proportion of the leaf cross section in the three stages (young, adult, senescent, Figure 2a–c and Table 1). This tissue was mainly located on the adaxial side and surrounding vascular bundles. Leaf tissue became more fragile with age, so empty spaces between spongy parenchyma and lower epidermis were only found in senescent leaves (Figure 2c). Cuticle was only apparent on the adaxial side and with a comparable thickness among leaf types: an average of 4.3 ± 0.1 μm (Figure 2d–f and Table 1). A few trichomes were present on the adaxial side of the leaf, while the abaxial side was densely covered by at least two different types of trichomes: glandular and non‐glandular (Figure 2e,f). Stomata were hardly found in cross‐sections, but always related to the abaxial stomatal crypts (hypostatic leaves with semi‐sunken stomata) (Figure 2g–i).

**TABLE 1:** Anatomical leaf‐traits obtained after analyses of images obtained under the optical microscope.

Leaf type	Cuticle thickness (μm)	Parenchyma area (% of leaf cross‐section)	Vascular bundle Ø (μm)
Young	4.17 ± 0.24 a	53.7 ± 0.6 a	152.4 ± 4.4 a
Adult	4.43 ± 0.25 a	58.6 ± 0.8 a	216.7 ± 5.2 a
Senescent	4.27 ± 0.22 a	15.9 ± 0.3 b	230.9 ± 5.7 b

*Note:* Average ± SE is shown (*n* = 6 biological replicates and 5 cross‐sections per leaf type). Statistical differences among leaf types are shown with different letters, *p* < 0.05.

### 3.2 Photosynthetic Pigments, Leaf Gas Exchange and Soluble Carbohydrate Content

**FIGURE 3:**
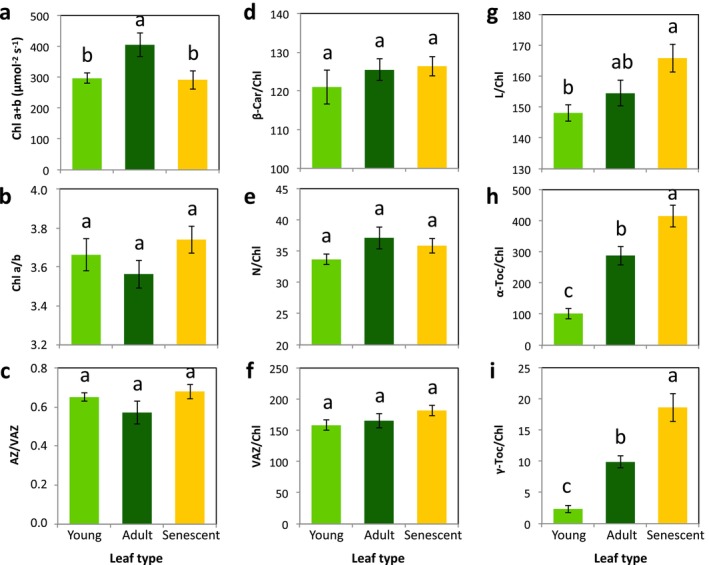
Leaf content of main photosynthetic pigments and tocopherols. Units are mmol mol^−1^ Chl for β‐carotene (β‐Car), lutein (L), xanthophyll cycle pigments (VAZ), neoxanthin (N), tocopherols (Toc), and mol mol^−1^ for the ratios chlorophyll a/b (Chl a/b) and de‐epoxidation of xanthophyll cycle pigments (AZ/VAZ). Data are means ± SE (*n* = 5) for each leaf type. Letters above bars indicate significant differences among leaf types (*p* < 0.05).

Leaf pigment and tocopherol composition, sampled at midday in spring, differed among leaf types (Figure 3). Adult leaves had the highest total chlorophyll (Chl a + b) content (405.1 ± 30.3 μmol m^−2^, Figure 3a), but all leaf types showed comparable Chl a/b (around 3.6, Figure 3b) and AZ/VAZ ratios (0.6 approximately, Figure 3c). Carotenoids (β‐carotene, neoxanthin (N) and VAZ) showed non‐significant differences (Figure 3d–f), while Lutein (L) and tocopherols (Toc) increased significantly with leaf age (Figure 3g,h,i). This increase in Toc content was remarkable: 4‐fold in the case of α‐Toc and 8‐fold in the case of γ‐Toc when comparing senescent vs. young leaves (Figure 3h,i).

**FIGURE 4:**
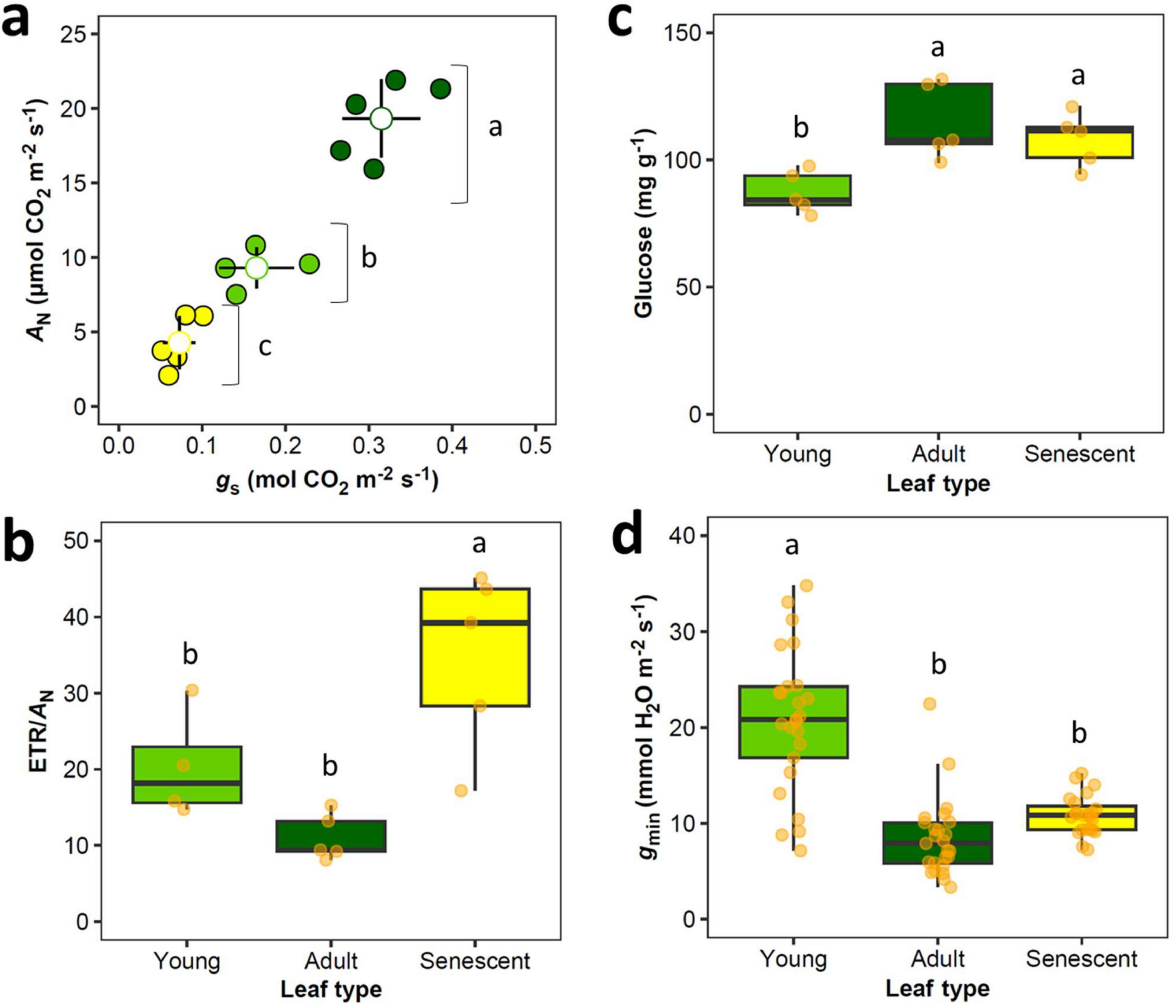
Gas exchange analyses and carbohydrate content in the three leaf types of *B. exstipulata*, measured in June 2021. (a) Net carbon assimilation (A_N_) versus stomatal conductance (g_S_). Close symbols are individual values, open symbols are average ± SE per leaf type. (b) Electron use efficiency for carbon assimilation (ETR/A_N_). (c) Glucose content per g^−1^ leaf DW (average ± SE). (d) Minimal conductance (cuticle conductance, g_min_). Different letters indicate significant differences among leaf types at *p* < 0.05 (*n* = 5 different individuals per leaf type, except for g_min_ where *n* = 20).

Average net carbon assimilation (A_N_) measured in late spring was 19.0 ± 0.6 μmol CO_2_ m^2^ s^−1^ for adult leaves (Figure 4) but it was significantly lower in young leaves (9.2 ± 0.7 μmol CO_2_ m^2^ s^−1^) and declined sharply at senescence (4.6 ± 0.4 μmol CO_2_ m^2^ s^−1^) (Figure 4a). Stomatal conductance (g_s_) correlated linearly with A_N_, being higher in adult (0.305 ± 0.014 mol H_2_O m^−2^ s^−1^) than in young (0.164 ± 0.013) and senescent (0.076 ± 0.005) leaves (Figure 4a). However, ETR was not coupled to assimilation (Figure 4b). Notably, the total soluble carbohydrate concentration, estimated as glucose equivalents, was comparable in adult and senescent leaves but significantly lower (22%) in young leaves (Figure 4c). Senescent leaves had the lowest electron use efficiency for carbon fixation (e.g., highest ETR/A_N_, Figure 4b). Leaf minimum conductance (g_min_) (which represents the cuticular plus the stomatal conductance when stomata are closed) was 8.4 ± 0.8 mmol H_2_O m^−2^ s^−1^ in adult leaves (Figure 4d). These values were similar in senescent leaves but significantly higher in young leaves (20.8 ± 1.5) (Figure 4d).

### 3.3 Loss of Leaf Capacity to Rehydrate and Xylem Vulnerability to Water Shortage

The loss of capacity to rehydrate was significantly higher in young and senescent than in adult leaves (Table 2). Young leaves were also able to rehydrate after more severe dehydration than adult and senescent leaves: RWC at PLRC_50_ was 17.7%, 45.1% and 41.3%, respectively. In terms of photochemical activity, however, adult leaves showed the highest capacity to recover: RWC at PLFCrh_10_ was 22.3% in adult leaves, 27.4% in young and 48.7% in senescent leaves. Young leaves only reached 50% PLCFrh at RWC as low as 8%. Thus, lower RWC was needed for a greater drop in the photochemical activity upon rehydration than in the rehydration capacity. Regarding leaf structure, young leaves had significantly lower LMA and higher saturated water content (SWC) than adult and senescent leaves (Table 3).

**TABLE 2:** Fitting and extracted parameters of the relationship between the relative water content (RWC) reached upon dehydration and the percentage loss of rehydration capacity (PLRC) or percentage loss of Fv/Fm recovery capacity upon rehydration (PLFC_rh_) in the three leaf types.

RWC vs. PRLC	Leaf type	Best adjustment	*R* ^2^	PLRC_10_ (%)	PLRC_50_ (%)
	Young	Sigmoidal	0.94	45.5	17.7
	Adult	Sigmoidal	0.96	57.9	45.1
	Senescent	Sigmoidal	0.97	62.4	41.3

RWC vs. PLFCrh	Leaf type	Best adjustment	*R* ^2^	PLFC_10_ (%)	PLFC_50_ (%)
	Young	Sigmoidal	0.99	27.4	—
	Adult	Sigmoidal	0.85	22.3	21.1
	Senescent	Sigmoidal	0.87	48.7	27.3

*Note: n* = 20 per leaf type, each leaf from different individual.

**TABLE 3:** Leaf traits.

Leaf type	LMA (mg cm^−2^)	SWCw (gH_2_O g^−1^ DW)	SWCa (gH_2_O cm^−2^)
Young	13.2 ± 0.63 a	2.60 ± 0.11 a	39.7 ± 3.25 a
Adult	17.5 ± 0.74 b	1.89 ± 0.05 b	37.9 ± 3.11 ab
Senescent	17.6 ± 0.81 b	1.92 ± 0.05 b	28.8 ± 4.21 b

*Note:* Average ± SE is shown (*n* = 6). When significant, differences among leaf types are shown with different letters, *p* < 0.05.

Abbreviations: LMA: leaf mass per area; SWCa, saturated water content on an area basis; SWCw, saturated water content (dry weight basis).

**FIGURE 5:**
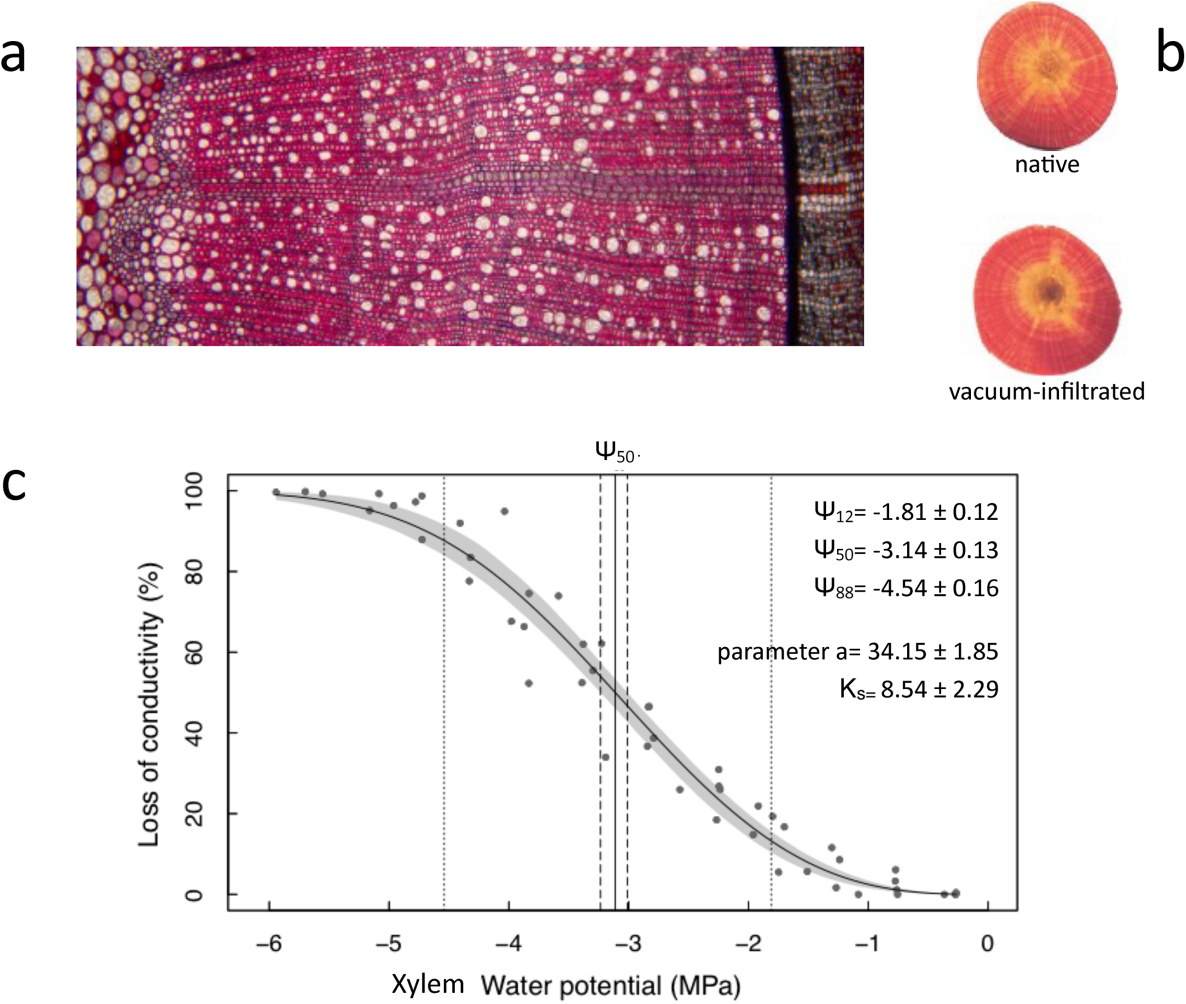
Xylem hydraulic properties. (a) Branch cross section of *B. exstipulata* stained with safranin and Astra blue under the microscope. (b) Cross‐section of *B. exstipulata* branch, showing virtually no cavitation (conductive xylem) in September 2021 (native) in comparison with vacuum‐infiltrated samples. (c) Percent loss of conductivity (PLC) versus water potential in *B. exstipulata* branches. The shaded area represents the 95% bootstrapped confidence interval for the fitted curve. From left to right, solid and dashed vertical lines indicate the water potentials at 88%, 50% and 12% PLC, respectively. For Ψ_50_ the ±confidence interval is additionally depicted. The inset shows the water potential (MPa) at 12%, 50%, and 88% loss of conductivity (Ψ_12_, Ψ_50_, Ψ_88_), Parameter *a* (related to the slope of the curve at the inflection point) and specific hydraulic conductivity (k_s_; × 10^−4^ m^2^ s^−1^ MPa^−1^) for *B. exstipulata*. Values given are mean ± SE (*n* = 5).

The xylem of *B. exstipulata* branches showed a diffuse‐porous anatomy. It was composed of tracheids and wider vessels plus parenchymatic radial and axial cells (Figure 5a). Samples collected at the end of summer (September 2021) did not show signs of loss of conductivity due to native embolism (i.e., staining did not differ between native and vacuum‐infiltrated samples) (Figure 5b). Branches from *B. exstipulata* showed characteristic sigmoidal vulnerability curves (Figure 5c). The threshold for 50% loss of conductivity (Ψ_50_) occurred at −2.99 ± 0.08 MPa (Figure 5, inset). The curve showed a relatively flat shape (see ‘Parameter a’ in Figure 5 inset), resulting in a wide Ψ_12_–Ψ_88_ range. The specific hydraulic conductivity of *B. exstipulata* branches (k_s_) was 8.5 ± 2.3 × 10^−4^ m^2^ s^−1^ MPa^−1^ (Figure 5c, inset).

#### 3.3.1 Leaf Performance Under Sub‐Zero Temperature

**FIGURE 6:**
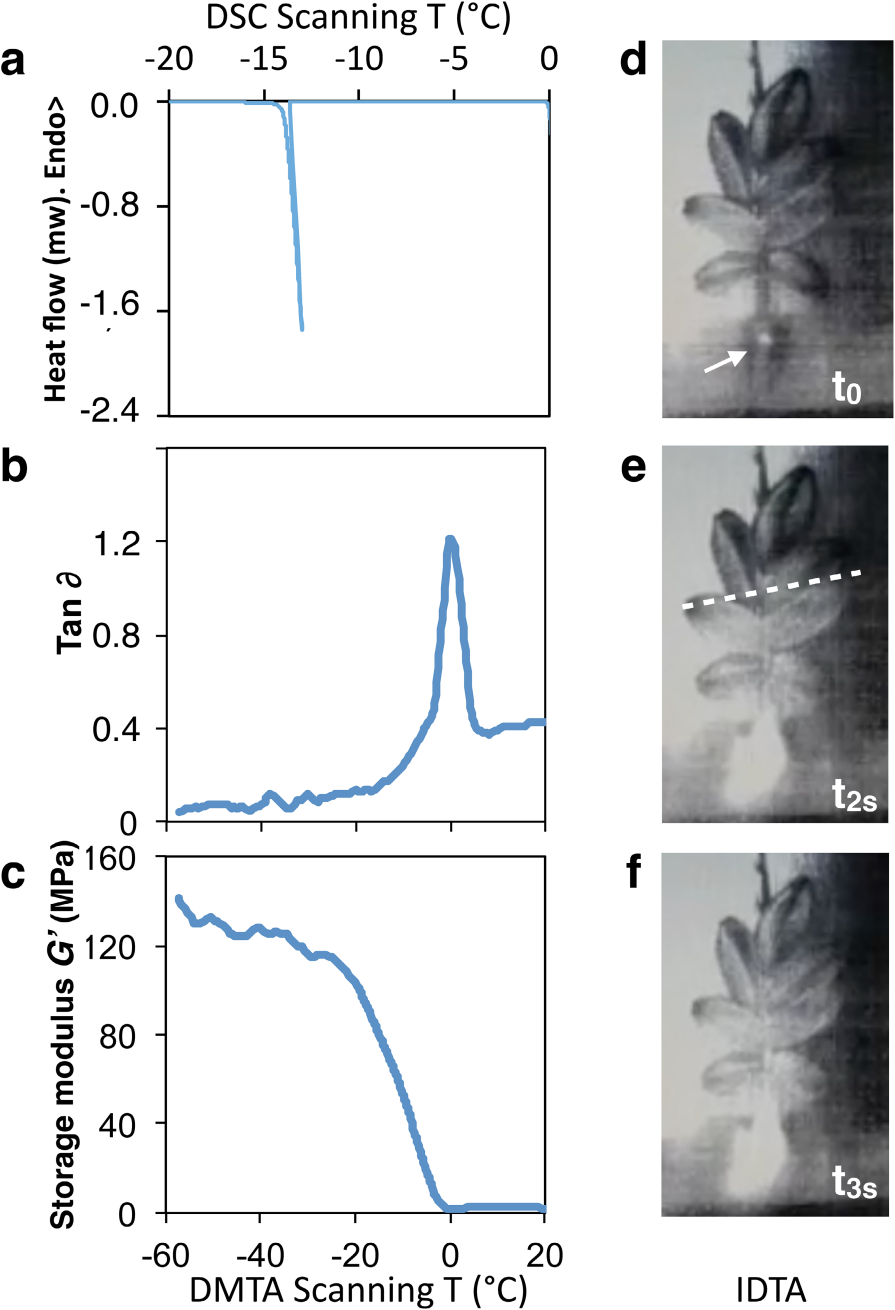
Biophysical analyses of adult winter leaves in response to sub‐zero temperatures. (a) Thermogram from differential scanning calorimetry (DSC) during a cooling. The downwards peak correspond to the exotherm process of ice formation inside the sample, and its onset was used for ice nucleation temperature (T_ice_) determination. Average ± SE obtained was −14.5°C ± 1.2°C (*n* = 5). One representative scan is shown. (b, c) Dynamic mechanical thermal analysis (DMTA) scans. (b) Delta tangent (Tan δ). (c) Storage modulus (*G*′). Glass transition temperature (T_g_), which is identified as a peak in the Tan δ and as a drop in the *G*′, represents the temperature below which viscosity increases greatly and the probability of enzymatic reactions decreases dramatically. Obtained average value ± SE (*n* = 3) was 0.0°C ± 1.2°C. One representative curve is shown for *G*′ and Tan δ (d–f) Ice propagation through the leaves obtained with an infrared differential thermal analyses (IDTA). Arrow and dashed lines indicate ice nucleation point and front, respectively.

Ice nucleation temperature (T_ice_) in turgid leaves of *B. exstipulata* acclimated to winter conditions was −14.5°C ± 1.2°C (Figure 6a). The dynamic mechanical thermal analyses (DMTA) of the same leaves provided information on their molecular mobility under cooling temperatures. As evidenced by the peak in the Tan δ, the glass transition temperature (T_g_) was 0.0°C ± 1.2°C (Figure 6b). As also observed in G', decreasing leaf temperature beyond zero dramatically increased cell viscosity and limited enzymatic reactions until becoming virtually impossible at ≤ −20°C, the temperature at which glass transition started (beginning of G' decrease during the DMTA heating scanning, Figure 6c). We did not find any anatomical barrier for ice propagation within the leaves when this was evaluated through the IDTA (Figure 6d–f). Ice nucleation during controlled freezing initiated in the leaf petiole (that was in touch with INA bacteria solution) at a leaf T of −5°C. Ice propagated smoothly from the base to the tip of the leaf until all leaflets were frozen in less than 5 s (ice propagated along the leaf at an average speed of 2 cm s^−1^).

**FIGURE 7:**
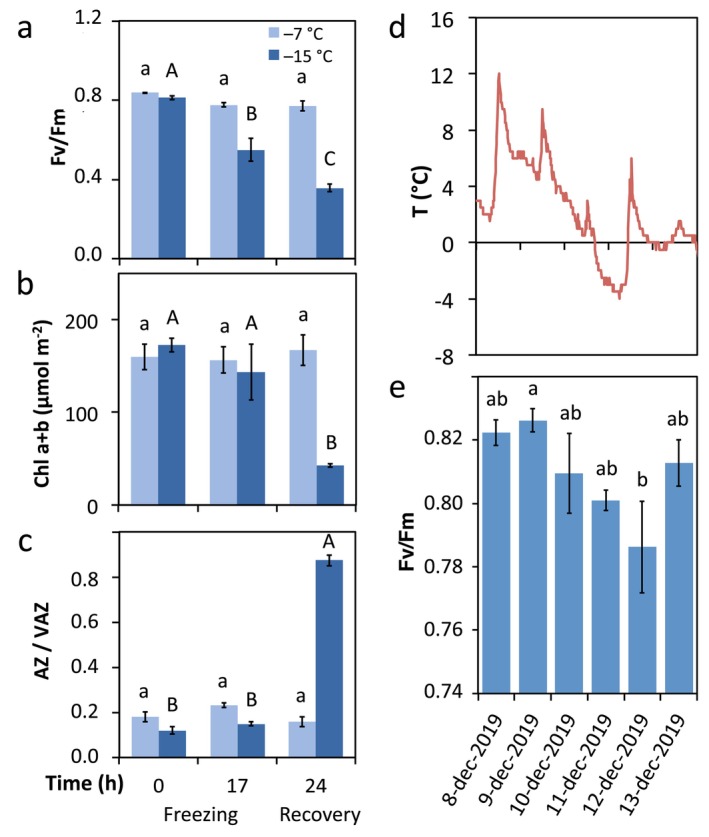
Physiological analyses of winter leaves in response to sub‐zero temperatures. (a–c) Changes in maximal photochemical efficiency and photosynthetic pigments obtained during controlled freezing down to −7°C or −15°C and subsequent recovery at +20°C (*n* = 6). Units for pigments are as in Figure 3. (d, e) Data from intact plants outdoors during winter. (d) Air temperature. (e) Evolution of predawn Fv/Fm these days (*n* = 4–8). All measurements were conducted in darkness. Bars show average ± SE. Letters denote significant differences at *p* < 0.05.

Freezing experiments performed under controlled conditions at a temperature just below (−15°C) or higher (−7°C) than the T_ice_ obtained by DSC revealed the capability of *B. exstipulata* to withstand sub‐zero temperatures (Figure 7). Interestingly, Fv/Fm was barely affected after 17 h at −7°C but it decreased significantly at −15°C (Figure 7a). Rewarming, however, revealed more marked differences between samples that had been subjected to −7°C (which kept their initial Fv/Fm values) and those that had been subjected to −15°C, which failed to recover, and even decreased further. The latter, additionally, showed significant destruction of chlorophylls during the first hours upon rewarming (Figure 7b) and exacerbated de‐epoxidation of the xanthophyll cycle pigments (Figure 7c). Monitoring of Fv/Fm during several winter days at predawn was in agreement with the lab experiments and revealed significant but very small (≤ 5%) decrease in Fv/Fm and only when night temperatures dropped below zero (Figure 7d,e). A complementary freezing experiment conducted at −9°C, −12°C and −18°C with young, adult and senescent leaves in December 2025 following the procedure of (Arzac et al. 2024; Castanyer‐Mallol et al. 2025), further reinforced this information: (1) after thawing, Fv/Fm dropped to 50% of the initial value irrespective of the leaf age and temperature tested and (2) T_ice_ was −13.5°C ± 0.5°C (Supporting Information: Data S2).

## 4 Discussion

### 4.1 Photosynthesis and Phenology Under Natural Conditions

To the best of our knowledge, this work constitutes the first physiological investigation conducted within the genus *Bencomia* (Rosaceae) and the first study on *B. exstipulata*, the only broadleaf tree living in the high elevation habitats that develop above the clouds in the slopes of Mt. Teide. Growing at relatively high elevation and in an arid ecosystem, three leaf traits of *B. exstipulata* were particularly remarkable: their unexpectedly short lifespan, their high net carbon assimilation rate, and their thin cuticle. According to the foliar habit classification provided by Kikuzawa and Lechowicz (2011), *B. exstipulata* is a ‘leaf exchanger’ species (leaf lifespan < 1 year, but the canopy contains functional leaves all along the year). The absence of significant difference in the non‐structural carbon concentration (glucose equivalents) we obtained when comparing adult vs senescent leaves and the lower concentration in young leaves, is in agreement with the results obtained in *Cistus laurifolius* (another Mediterranean ‘leaf exchanger’), and does not support carbohydrate demand by new leaves as the trigger for leaf replacement (Milla et al. 2007). The A_N_ of 19.0 ± 0.6 μmol CO_2_ m^2^ s^−1^ obtained in the adult leaves of *B. exstipulata* was within the range of optimal values obtained for other woody species that inhabit the same summit scrub at Teide Mountain (19 to 34 μmol CO_2_ m^2^ s^−1^) (Perera‐Castro et al. 2025), 10‐fold higher than the optimal values obtained by García‐Núñez et al. (2004) for a close relative of *B. exstipulata*, original from the Andes: *Polylepis tarapacana* (2–3 μmol CO_2_ m^2^ s^−1^) and 1.6‐fold higher than the maximal A_N_ measured in this later species by García‐Plazaola et al. (2015). *In B. exstipulata*, the highest proportion of carbon assimilation was conducted by adult leaves that also contained higher Chl content per leaf area. The ratio ETR/A_N_ increased dramatically with leaf senescence, indicating a decoupling between photochemical and assimilation processes and lowered yield (Perera‐Castro and Flexas 2023), and a high proportion of electrons was directed to processes other than carbon assimilation, tentatively to the formation of reactive oxygen species (ROS). Although ROS were not directly measured, we quantified lipophilic antioxidants and transition from mature to senescent leaves was accompanied by a dramatic increase on α‐ and γ‐tocopherol contents. This pattern of age‐dependent changes in tocopherol is in agreement with previous studies (Hormaetxe et al. 2004; Lizarazo et al. 2010). The rapid turnover of the leaves may be thus related with a high photoprotective demand (e.g., the obtained VAZ/Chl values above 150 mmol mol^−1^ are 3‐fold higher than the most common value for terrestrial plants ≈50 mmol mol^−1^ (Esteban et al. 2015)) and can additionally represent a functional strategy under low‐water and low‐nutrient availability conditions (Sardans and Peñuelas 2013). Overall, it seems that *B. exstipulata* carbon gain relies mostly on adult leaves that conduct maximum photosynthesis rate during approximately 2–3‐months in the middle of their lifespan, and are quickly replaced, all along the year, very likely due to a quick photooxidative aging.

### 4.2 Water Relations

Despite the relatively arid conditions of the environment, including average RH around 30% and low precipitation (i.e., 213.6 mm in 2019; 233.8 mm in 2020; and 91.8 mm in 2021 (Teide National Park Reports 2019–2021)), analysed traits surprisingly indicated neither clear adaptations to drought nor signs of drought stress for *B. exstipulata*. Thus, high g_s_, thin cuticle, high g_min_, high photochemical resistance to leaf dehydration, and high xylem vulnerability to embolisation (but no native embolism) were obtained.

The g_s_ was high in *B. exstipulata* (up to 0.4 mol H_2_O m^−2^ s^−1^ in adult leaves) in spring. These values are at least 4‐times higher than in well‐watered plants of other mediterranean shrubs, that is, *Cistus albidus* (Lorente et al. 2021); *Rosmarinus officinalis* , *Nerium oleander* , or *Rhamnus lycioides* among others (Moreno‐Gutiérrez et al. 2012); and also, much higher than those obtained under natural conditions in its relative *P. tarapacana* (García‐Núñez et al. 2004). It seems, then, that high g_s_ rates are kept provided enough water is available in the soil, despite the small and sunken location of the abaxial stomata, and the hairy abaxial side of the leaves. Remarkably, a very thin cuticle (almost absent in the abaxial side of the leaves) was observed. Thick cuticles could be disadvantageous in environments characterised by intense winds and recurrent frosts, either for biomechanical or economic reasons (Li et al. 2025). Additionally, it has been established that prolonged exposure to wind can raise the conductance of the cuticle due to abrasion (Rogge et al. 1993). Cuticular plus stomatal conductance (when stomata are closed) constitutes the minimum conductance (g_min_). Interestingly, in spite of sunk stomata and very hairy leaves, g_min_ of *B. exstipulata* was very high (up to 20 mmol m^−2^ s^−1^ in young leaves, and near 10 in adult and senescent leaves) if compared to other Rosaceae (around 5 mmol m^−2^ s^−1^, Duursma et al. 2019) and within the range but in the highest values of Alpine shrubs sampled at similar elevation than *B. exstipulata* (1.37 to 8.97 mmol m^−2^ s^−1^, Musso et al. 2025). This added to the very high g_s_ supports the idea of a not very tight regulation of water loss at the leaf level. Additionally, within a single species g_min_ can be very plastic, being significantly higher in well‐watered than in drought‐stressed plants (Duursma et al. 2019). Indeed, *Rosa* spp. grown at different evaporative demands are able to change stomatal anatomy and showed altered closure during desiccation leading to changes in g_min_, without changes in cuticle (Fanourakis et al. 2013). It seems either that (1) the thin cuticle of *B. exstipulata* is relatively permeable or (2) that its stomata remain incompletely closed upon drying conditions, leading to a relatively high g_min_. This indicates a well‐watered condition at least during leaf development. All these observations identify *B. exstipulata* as a water‐spender species according to Levitt (1980), capable of maintaining high gas‐exchange rates whenever soil water is available, despite the low rainfall characteristic of its environment. This is somewhat counterintuitive, since one might expect such a hostile environment to be inhabited by highly resilient species. Similar behaviour has been observed in one of the dominant species of the summit‐shrub ecosystem at Teide National Park, namely *Spartocytisus supranubius* (González‐Rodríguez et al. 2017). The water‐spender behaviour of both species is in agreement with a potential decoupling between recent rainfall and actual water availability in the soil. We hypothesise that the mulching effect of the pyroclastic materials that cover the soil may warrant water availability in the root zone by preventing excessive evaporation, as it has been proved to be very effective for soil water conservation under arid conditions (Tejedor et al. 2003).

We additionally analysed the vulnerability to water shortage of the leaves through the estimation of loss of rehydration capacity (LRC) and of the xylem through vulnerability curves. The loss of rehydration capacity of 50% (PLRC_50_) of completely developed leaves (adult and senescent) occurred around 50% of RWC in *B. exstipulata*. This is higher than the values obtained for other species of the Rosaceae family growing under Mediterranean climate, such as *Cerocarpus betuloides* or *Heteromeles arbutifolia* (≈37% RWC), and also higher than in some Rosaceae species from temperate forests such as *Rhadphiolepis indica* : 40% RWC (Trueba et al. 2019). Thus, less severe leaf dehydration is needed to lose 50% of rehydration capacity in *B. exstipulata* leaves. More remarkably, completely developed leaves lost 50% of photochemical efficiency capacity after a dehydration/rehydration cycle at much higher RWCs 22%–48% than any of these aforementioned 3 Rosaceae species (5%–15% RWC at PLCF_50_). This means that photochemical efficiency is considerably more vulnerable to dehydration/rehydration in *B. exstipulata* than in the other species of the same family. Its xylem vulnerability to embolism was also relatively high: Ψ_50_ occurred at around −3 MPa in comparison with some drought‐tolerant Mediterranean species, such as *Rhamnus alaternus* (Ψ_50_–6 MPa) or in comparison with the global average of shrubs from mountain regions, including all climates (−4.2 MPa) (Musso et al. 2025). *B. exstipulata* values were, however, in the range of other woody species from the Mediterranean Basin or from California (−2 to −4 MPa) (El Aou‐Ouad et al. 2017; Fickle et al. 2023) or Alpine shrubs growing at similar elevation (−2 to −3 MPa, Ganthaler and Mayr 2021). Despite the comparably high vulnerability, no native embolism was detected at the end of summer (Figure 5), indicating that branch water potentials in *B. exstipulata* never reached critical values, or alternatively, that some repair is possible if happened. As an evidence in the former sense: leaf Ψ_w_ measured at predawn on the 17–21st Feb 2020 (coinciding with a severe Calima event of dry and sandy windy conditions (Le Blancq 2020)) was −0.5 ± 0.09 MPa, and A_N_ these days was 15 ± 1.4 μmol CO_2_ m^2^ s^−1^ (data not shown). The efficiency of the xylem water transport was with 8.54 × 10^−4^ m^2^ s^−1^ MPa^−1^ below the average for shrub species in the Xylem Functional Trait Database (16.6 ± 0.1 × 10^−4^ m^2^ s^−1^ MPa^−1^; Gleason et al. 2016), but higher than in Alpine shrubs within the genus *Rhododendron* (between 1.92 and 3.45 × 10^−4^ m^2^ s^−1^ MPa^−1^, Mayr et al. (2010)). These two traits (high PLCF_50_ and Ψ_50_) are in agreement with a usual high‐water availability for *B. exstipulata*.

The presence of filiform and glandular trichomes on the abaxial surface of *B. exstipulata* likely contributes to its adjustment to the semi‐arid, high‐radiation conditions of the Teide summit scrub by modulating leaf water and energy balance. In semi‐arid shrubs, leaf hairs have been shown to reduce leaf temperature, limit excessive transpiration and improve carbon gain under high irradiance, as demonstrated for xeromorphic species such as *Wunderlichia azulensis* , where the presence of dense trichomes conferred clear physiological advantages under drought and heat stress (Gomes Trindade et al. 2022). Experimental work in xeric and semi‐arid plants further indicates that trichomes can facilitate drought acclimation by increasing leaf reflectance, thickening the effective boundary layer and reducing overheating, while their density often increases under water limitation, as reported for *Shepherdia × utahensis* and other drought‐tolerant species (Chen et al. 2022). In addition, leaf trichomes can enhance water acquisition from non‐rainfall inputs, such as dew, as shown for *Caragana korshinskii* , where trichome‐mediated dew absorption helped maintain leaf water potential and hydraulic conductivity under severe soil drought (Waseem et al. 2021). This would be an aspect to test in the future since no evidence of native embolism was found in the xylem, and water absorption by leaves could alleviate severe drought events.

All things considered, and despite the apparent aridity of the environment, *B. exstipulata* does not show typical adaptations to drought (except for leaf trichomes and sunken stomata). By contrast, compiled evidence indicates a balanced water supply and no occurrence of very low internal water potentials, enabling the plants to optimise gas exchange and reduce cost‐intensive investments in their structure. We can only hypothesise that the species has access to ground water, what prevents embolisation, although use of ‘occult precipitation’ form from fog or dew should also be addressed in future studies. Overall, the species seems relatively independent of changes in precipitation and well adapted to the harsh conditions of the summit scrub, but may find strong limitations on its distributional range, likely dependent on local sources of water. This may explain its rarity—not just herbivore pressure—or why the early expeditions of Humboldt and Bonpland failed to find the species.

### 4.3 Cold‐Facing Strategy

Our results support a freezing avoidance strategy for *B. exstipulata* in agreement with other species of the genus, such as *B. sericea* (Squeo et al. 1991). Interestingly, vitrification of the photosynthetic tissue (which, to the best of our knowledge, has been measured in a woody species for the first time in this work) already started at 0°C. This is a relatively high temperature compared with that of ice‐tolerant species (Fernández‐Marín, García‐Plazaola, et al. 2018; Fernández‐Marín, Neuner, et al. 2018). This already high viscosity of photosynthetic tissue at a relatively warm temperature may explain why no enzymatic accumulation of protective zeaxanthin was observed upon freezing, in contrast to other previously tested ice‐tolerant species (Fernández‐Marín, García‐Plazaola, et al. 2018; Fernández‐Marín, Neuner, et al. 2018; Fernández‐Marín et al. 2021). Accordingly, significant signs of chronic winter photoinhibition were absent, as was also the case for species of *Polylepis* in the Andes (García‐Plazaola et al. 2015), even at subzero temperatures down to −7°C. Significant reductions in the predawn Fv/Fm measured outdoors occurred only after two consecutive nights of sub‐zero temperatures, but these were reversible during subsequent warmer nights. This indicates a small and dynamic component of winter photoinhibition in the species. Strong and irreversible reductions were only obtained below the T_ice_ (e.g., at −15°C). While our obtained T_ice_ is low, and it must be considered that detached leaves can show artificially decreased ice nucleation temperature when compare to native conditions, T_ice_ of −3°C have been obtained with other species using this technique (Fernández‐Marín, Neuner, et al. 2018) Additionally, by using a different approach with adult plants, the leaves of the three ages: young, adult and senescent, during winter 2025, we obtained the same range of values: −13.9°C to −14.9°C without significant differences among leaf ages (Supporting Information S2).

Anatomical barriers for ice propagation were not found (Bertel et al. 2021). Lethal temperature for the leaves was set in the range of −7°C to −9°C. These are a few °C lower than the minimum air temperature usually recorded at El Portillo (de Canarias and de Tenerife 2019, 2020, 2021), but are within the range of autumn to spring minimum temperatures in many areas of its potential distribution within Teide National Park (López Díez et al. 2021; Alday Echechipia et al. 2025). Overall, the data support a limited capacity of *B. exstipulata* leaves to strongly down‐regulate photochemical efficiency as a photoprotective strategy in response to sub‐zero temperatures, a supercooling strategy, and a consequent vulnerability against strong freezing events.

### 4.4 Ecophysiological Perspective on a Climate Change Framework

In the context of global climate change, oceanic high‐mountain ecosystems such as Teide National Park are undergoing rapid shifts that threaten endemic flora. Observed and projected climate trends reveal an alarming scenario: a warming rate of 1.7°C ± 0.6°C per decade between 1975 and 2015 (Martin‐Esquivel and Perez‐Gonzalez 2019) and pronounced nighttime warming (Correa et al. 2025), all of which have an exacerbating effect on plant water stress (Expósito et al. 2015). Related to this, increasingly dry conditions and shorter wet seasons are already being experienced (Martín‐Esquivel et al. 2021), and all these trends are projected to intensify (Cruz‐Pérez et al. 2025). In light of the obtained results, *B. exstipulata* is very likely a vulnerable species against drought events, and future studies are encouraged to shed further light on its hydraulic limits, water sources (deep soil resources? occult precipitation included?), and/or its capability to repair xylem embolism. Another consequence of warming temperatures at Teide Mountain is the upward migration of plant species in an attempt to avoid extremely high maximum temperatures (González‐Rodríguez et al. 2021; Renner et al. 2023). This upward migration is, however, not an option for all species due to the wider thermal amplitude and the minimum temperatures acting as limiting conditions. Thus, upward migration may prevent *B. exstipulata* from too warm average temperatures but may expose it to too low minimum temperatures beyond its tolerance. Hence, given that *B. exstipulata* thrives above the inversion layer in an already thermally extreme habitat, and with a supercooling strategy, the species may be approaching its physiological limit for cold resistance, too.

## 5 Concluding Remarks

The endemic shrub *Bencomia exstipulata*, with a very limited distribution at about 2000 m a.s.l. on the Canary Islands, is characterised by a high leaf turnover, high gas exchange rates plus comparable high g_min_, high vulnerability to xylem cavitation and rapid loss of leaf rehydration capability upon dehydration, which is surprising for the relatively arid conditions of its native environment. Its cold strategy is freeze‐avoidance, relying on supercooling and early vitrification, what keeps its leaves safe at temperatures moderately below zero but makes them sensitive to severe frost events. This comprehensive ecophysiological characterisation can help to better understand the ecological amplitude of the species and to optimise future management actions. In light of the current results, severe drought and, to lesser extent, extreme freezing events, could compromise its survival. The orography and climatic heterogeneity of the Teide National Park if considered by managers (Martín‐Esquivel et al. 2021) may offer microclimatic refugia for its future persistence.

## Author Contributions

B.F.‐M. designed the work conducted the data analyses and wrote the ms. A.V.P.‐C. conducted the photosynthesis and g_min_ measurements. P.B.‐G. conducted the phenological analyses. G.N., J.M.L. and J.H. participated in the analyses under subzero temperature. J.I.G.‐P. and M.I.A. conducted pigment analyses. M.A.R.‐M. conducted anatomical analyses. A.G. and S.M. conducted xylem analyses. J.P. and A.M.G.‐R. conducted loss of rehydration capacity analyses. All authors contributed to and approved the final version of the manuscript.

## Funding

This research was funded through the following research grants: ECOSCAN grant no. 2751/2021 funded by the ‘Organismo Autónomo de Parques Nacionales, MITECO’; POPEYE grant no. PID2022‐139455NB‐C32 funded by MCIN/AEI/https://doi.org/10.13039/501100011033 and by ‘ERDF A way of making Europe’ by the European Union; Austrian Science Fund (FWF), project ‘The Future of Mountain Forests’, grant‐doi: https://doi.org/10.55776/DOC171. M.I.A. enjoyed a pre‐doctoral grant from the Basque Government. B.F.‐M. enjoyed the RYC2021‐031321‐I grant funded by MCIN/AEI/https://doi.org/10.13039/501100011033 and by the European Union NextGenerationEU/PRTR.

## Supporting information


**Data S1:** Details on the methodology for staining conductive xylem área in *Bencomia exstipulata* branches.
**Data S2:** Data from a complementary freezing test conducted with young, adult and senescent leaves in December 2025.
**Data S3:** Summary table of main parameters and number of replicates.

## Data Availability

The data that supports the findings of this study are available in the Supporting Information of this article.
