# Supplementary material for: Drought and Freezing Compromise Woody‐Plant Functioning in High Mountain Ecosystems Under Mediterranean Climate: The Case of Bencomia exstipulata
Source: Physiol Plant. 2026 Jul 3;178(4):e70965. doi: 10.1111/ppl.70965 (PMC13330751; doi:10.1111/ppl.70965)
Supplement: Supplementary file 1 — Data S1: Details on the methodology for staining conductive xylem área in Bencomia exstipulata branches. Data S2: Data from a complementary freezing test conducted with young, adult and senescent leaves in December 2025. Data S3: Summary table of main parameters and number of replicates. [file PPL-178-e70965-s001.pdf]

**SUPPLEMENTARY DATA**

**Article Title**

**Drought and freezing compromise woody-plant functioning in high mountain ecosystems under Mediterranean climate: the case of *Bencomia exstipulata*.**

**Authors**

**Fernández-Marín B**[1,2], Perera-Castro AV[2,3], Brito-Gutiérrez P[2,4], Arzac MI[1], Díaz Peña FJ[5], Ganthaler A[6], García-Rodríguez C[2], Laza JM[7], Luis Díaz V[2], Hidalgo J[1], Mayr S[6], Neuner G[6], Ruiz-Medina MA[2], García-Plazaola JI[1], Puértolas J[2], González-Rodríguez AM[2]

**Supplementary Data S1.** Details on the methodology for staining conductive xylem area in *Bencomia exstipulata* branches.

From the base of three branches, two segments (length ca. 3cm) were cut and debarked under water. One sample was vacuum infiltrated for three hours in distilled, filtered (0.22 µm) and degassed water containing 0.005% (vol/vol) Micropur Forte MF 100F (Katadyn Products) to prevent microbial growth (Beikircher and Mayr, 2008), the other sample was kept wet until staining (native samples). Afterwards, segments were trimmed at both ends (submersed in the solution) with a sharp carving knife (Beikircher and Mayr, 2016), before the segments were sealed in a hydraulic system (modified after Sperry et al., 1988) connected to a reservoir filled with 0.1% (wt/vol) filtered (0.22 µm) safranin solution (Ganthaler and Mayr 2021). The pressure was set to 5 kPa, and samples remained connected until the outflow was deeply red-stained. After a drying period of 30 min at room temperature, cross sections were made from the middle part of the stained stem sections with the carving knife (discs of ca. 1 mm) and photos taken with a binocular stereo microscope (Olympus SZ61; Olympus Austria) interfaced with a digital camera (ProgRes CT3, Jenoptik).

**Supplementary Data S2.** Data from a complementary freezing test conducted with young, adult and senescent leaves in December 2025.

Leaves of the three ages were collected from 6 individuals at evening in the field and preserved at +4°C, 100% RH and darkness until freezing experiment. Whole leaves were cooled inside a freezer and change in temperature monitored with a temperature recorder (BTM4208SD, Lutron Instruments) with K-type thermocouples at a rate of 1 record per second to estimate the ice nucleation temperature. The table on the right shows the average ± SE of the ice nucleation temperature per leaf type. In parallel, 6 replicates per leaf age were used during a controlled cooling and thawing treatment following the method described in Arzac et al. (2024) and Castanyer-Mallol et al. (2025). The Fv/Fm was measured individually in each sample previous to freezing and after 12h of recovery upon thawing. Right panel shows the % to initial Fv/Fm as average per leaf age and target temperature tested (± SE).

| Leaf type | $T_{ice}$ (°C) |
|---|---|
| Young | -13.9 ± 0.5 |
| Adult | -12.9 ± 0.3 |
| Senescent | -14.9 ± 0.3 |
| Average | -13.4 ± 0.5 |

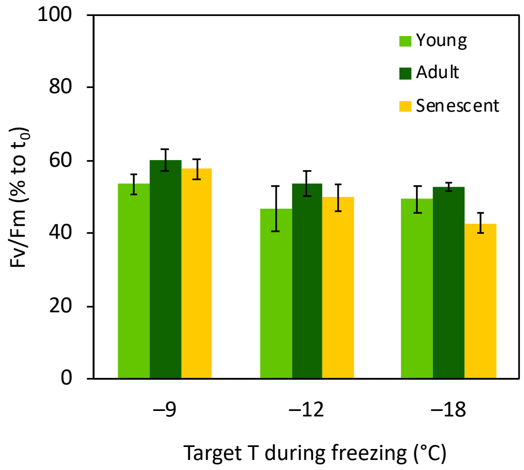

**Supplementary Data S3.** Summary of parameters, units and number of biological replicates used throughout the ms.

| Category | Parameter | Unit | n (plants) | Method | Figure/Table |
|---|---|---|---|---|---|
| **Leaf phenology** | Leaf number per branch | count | 21 | Field monitoring | Fig. 1 |
| | Leaf area | $cm^2$ | 21 | Image analysis (ImageJ) | Fig. 1 |
| | Leaf lifespan | months | 21 | Field observation | Fig. 1 |
| | Leaf developmental stages | categorical | 21 | Visual classification | Fig. 1 |
| **Leaf anatomy** | Cuticle thickness | µm | 6 | Light microscopy | Fig. 2; Table 1 |
| | Parenchyma proportion | % | 6 | Light microscopy | Fig. 2; Table 1 |
| | Bundle sheath diameter | µm | 6 | Light microscopy | Table 1 |
| | Leaf mass per area (LMA) | $g\ m^{-2}$ | 6 | Gravimetric | Table 3 |
| | Saturated water content on a dry weight basis (SWCw) | $gH_2O\ g^{-1}$ DW | 6 | Gravimetric | Table 3 |
| | saturated water content on an area basis (SWCa) | $gH_2O\ cm^{-2}$ | 6 | Gravimetric | Table 3 |
| **Pigments & metabolites** | Total Chlorophyll (Chl) | $\mu mol\ m^{-2}$ | 5 | HPLC | Fig. 3 |
| | Metabolites per Chl ($\beta$-Carotene, Neoxanthin, VAZ, Lutein, Tocopherols (α, γ) | $mmol\ mol^{-1}$ Chl | 5 | HPLC | Fig. 3 |
| | Ratios (AZ/VAZ; Chla/B | $mol\ mol^{-1}$ | 5 | HPLC | Fig. 3 |
| | Glucose | $mg\ g^{-1}$ DW | 5 | Spectrophotometry (anthrone) | Fig. 4 |
| **Gas exchange** | Net assimilation ($A_N$) | $\mu mol\ CO_2\ m^{-2}\ s^{-1}$ | 5 | IRGA | Fig. 4 |
| | Stomatal conductance ($g_s$) | $mol\ H_2O\ m^{-2}\ s^{-1}$ | 5 | IRGA | Fig. 4 |
| | Electron transport efficiency (ETR) | $\mu mol$ e- $m^{-2}\ s^{-1}$ | 5 | Chl Fluorometer | Fig. 4 |
| | Minimum conductance ($g_{min}$) | $mmol\ H_2O\ m^{-2}\ s^{-1}$ | 5 leaves/type | Gravimetric + VPD calculation | Fig. 4 |
| **Leaf water relations** | Relative water content (RWC) | % | 20 | Gravimetric | Table 2 |
| | Percentage Loss of rehydration capacity (PLRC) | % | 20 | Dehydration–rehydration assay | Table 2 |

| Category | Parameter | Unit | n (plants) | Method | Figure/Table |
|---|---|---|---|---|---|
| | Perentage Loss of Fv/Fm recovery capacity unpon rehydration ($PLFC_{rh}$)) | % | 20 | Chl fluorometer | Table 2 |
| | Thresholds ($PLRC_{50}$, $PLCF_{50}$) | % | derived | Model fitting | Table 2 |
| **Xylem hydraulics** | Hydraulic conductance ($K_h$) | $m^3\ s^{-1}\ MPa^{-1}$ | 5 | Cavitron | Fig. 5 |
| | Percentage loss of conductivity (PLC) | % | 5 | Cavitron | Fig. 5 |
| | Water potential at PLC ($\Psi_{12}$, $\Psi_{50}$, $\Psi_{88}$) | MPa | 5 | Weibull fit | Fig. 5 |
| | Specific conductivity ($k_s$) | $m^2\ s^{-1}\ MPa^{-1}$ | 5 | Derived from kh | Fig. 5 |
| | Native embolism | qualitative | 5 | Safranin staining | Fig. 5 |
| **Freezing (physical)** | Ice nucleation temperature ($T_{ice}$) | °C | 5 | DSC | Fig. 6 |
| | Glass transition temperature ($T_g$) | °C | 3 | DMTA (from Tand δ) | Fig. 6 |
| | Viscoelastic properties storage and loss modulus and (G', G'') | MPa | 3 | DMTA | Fig. 6 |
| **Freezing (physiology)** | Fv/Fm (controlled freezing) | unitless | 6 | Chl Fluorometer | Fig. 7 |
| | Pigments (controlled freezing) | $\mu mol\ m^{-2}$ | 6 | HPLC | Fig. 7 |
| | Fv/Fm (outdoor predawn) | unitless | 8 | Chl Fluorometer | Fig. 7 |
| | Ice propagation through the leaf | qualitative | 6 | Infrared thermography (IDTA) | Fig. 6 |
